# Molecular mechanisms of resistance to tyrosine kinase inhibitor in clear cell renal cell carcinoma

**DOI:** 10.1111/iju.15042

**Published:** 2022-09-19

**Authors:** Yohei Sekino, Jun Teishima, Gangning Liang, Nobuyuki Hinata

**Affiliations:** ^1^ Department of Urology, Graduate School of Biomedical and Health Sciences Hiroshima University Hiroshima Japan; ^2^ Department of Urology, USC Norris Comprehensive Cancer Center University of Southern California Los Angeles California USA

**Keywords:** drug resistance, metastatic renal cell carcinoma, molecular mechanism, tyrosine kinase inhibitors

## Abstract

Clear cell renal cell carcinoma (ccRCC) is the most common subtype of renal cell carcinoma (RCC). Loss of von Hippel–Lindau tumor suppressor gene is frequently observed in ccRCC and increases the expression of hypoxia‐inducible factors and their targets, including epidermal growth factor, vascular endothelial growth factor, and platelet‐derived growth factor. Tyrosine kinase inhibitors (TKIs) offer a survival benefit in metastatic renal cell carcinoma (mRCC). Recently, immune checkpoint inhibitors have been introduced in mRCC. Combination therapy with TKIs and immune checkpoint inhibitors significantly improved patient outcomes. Therefore, TKIs still play an essential role in mRCC treatment. However, the clinical utility of TKIs is compromised when primary and acquired resistance are encountered. The mechanism of resistance to TKI is not fully elucidated. Here, we comprehensively reviewed the molecular mechanisms of resistance to TKIs and a potential strategy to overcome this resistance. We outlined the involvement of angiogenesis, non‐angiogenesis, epithelial‐mesenchymal transition, activating bypass pathways, lysosomal sequestration, non‐coding RNAs, epigenetic modifications and tumor microenvironment factors in the resistance to TKIs. Deep insight into the molecular mechanisms of resistance to TKIs will help to better understand the biology of RCC and can ultimately help in the development of more effective therapies.

**Abbreviations & Acronyms**
ABC: ATP‐binding cassette
Ang2: angiopoietin‐2
CAF: cancer‐associated fibroblast
ccRCC: clear cell renal cell carcinoma
Dll4: notch ligand delta‐like 4
EMT: epithelial‐mesenchymal transition
EZH2: enhancer of zeste homologue 2
FGF: fibroblast growth factor
FGFR: fibroblast growth factor receptor
G‐CSF: granulocyte colony stimulating factor
HGF: hepatocyte growth factor
HIF: hypoxia‐inducible factor
IL: interleukin
MDSC: myeloid‐derived suppressor cells
MET: mesenchymal‐epithelial transition
miRNA: micro RNA
mRCC: metastatic renal cell carcinoma
PDGF: platelet‐derived growth factor
RCC: renal cell carcinoma
SLC: solute carrier
TAM: tumor‐associated macrophage
TEC: tumor endothelial cell
TKI: tyrosine kinase inhibitor
TME: tumor microenvironment
VEGF: vascular endothelial growth factor
VM: vascular mimicry

## INTRODUCTION

Renal cell carcinoma (RCC) represents around 3% of all cancers in adults,^1^ and there were more than 400 000 new cases worldwide in 2020.^1^ The cumulative risk of RCC in Japan is 0.6% compared to 1.5% in other developed countries.^2^ RCC accounts for approximately 90% of all renal tumors,^3^ of which clear cell renal cell carcinoma (ccRCC) is the most frequent (75%–80%). Papillary RCC and chromophobe RCC represent the most common remaining histologic subtypes with incidences of 7%–14% and 6%–11%, respectively.^4^ More than 30% of patients were diagnosed as having metastatic RCC (mRCC), which is a lethal disease with a 5‐year rate of survival of around only 10%.^5^, ^6^

Historically, the treatment of patients with mRCC has been limited because chemotherapy and radiation therapy are largely ineffective.^7^ Cytokine‐based immunotherapies (interleukin [IL]2, interferon‐alfa) benefited only a small subset of patients.^8^ Angiogenesis plays a pivotal role in the biology of ccRCC.^9^ Loss of the von Hippel–Lindau (VHL) tumor suppressor gene is observed in about 90% of ccRCC cases.^10^ Loss of this VHL gene activates the angiogenesis pathway, including epidermal growth factor, vascular endothelial growth factor (VEGF), and platelet‐derived growth factor (PDGF), through hypoxia‐inducible factors (HIF1 and HIF2).^11^ VEGF, which binds to tyrosine kinase receptors, is the essential growth factor involved in angiogenesis in ccRCC.^3^ The increased expression of VEGF helps to explain the hypervascularity of ccRCC and the development of therapies that specifically target the VEGF pathway in ccRCC. Over the last decade, treatment for mRCC has focused on targeting VEGF signaling with tyrosine kinase receptor inhibitors (TKIs). Various TKIs have shown considerable efficacy in ccRCC.^12^ According to the NCCN guidelines, several TKIs are clinically in use, such as sunitinib, sorafenib, pazopanib, cabozantinib, axitinib, lenvatinib, and tivozanib or monoclonal antibodies such as bevacizumab.^13^ In addition to the anti‐angiogenic roles of TKIs, recent studies showed the immunomodulator roles of TKIs in RCC.^14^, ^15^, ^16^ Indeed, combination therapy with TKIs and an immune checkpoint inhibitor has improved overall survival in mRCC.^17^ These findings indicate that TKIs still play an essential role in RCC treatment. Therefore, clarifying the mechanisms of TKI resistance is an urgent need.

In this review, we aimed to summarize the key molecular mechanisms behind the resistance to TKIs in ccRCC (Table 1) as well as the basis for the development of new drugs to overcome this resistance.

**TABLE 1:** Mechanism of resistance to tyrosine kinase inhibitors in renal cell carcinoma

Pathway	References
1. Angiogenic pathways	21, 23, 24, 25
2. Non‐angiogenic pathways	30, 31, 32, 33
3. Epithelial‐mesenchymal transition	36, 38, 39, 40, 41
4. Activating bypass pathway	
4.1. Cytokines	45, 46, 47, 48, 49, 50, 52, 53, 54, 56
4.2. PTEN/PI3K	62, 63, 64, 65
4.3. FGF/FGFR	70, 72
4.4. Axl/GAS6	75, 80, 81
4.5. HGF/MET	80, 81, 85, 86
4.6. Angiopoietin/Tie	90, 92, 93
5. Transporters	97, 98, 99, 100, 101, 102, 103, 104
6. Lysosomal sequestration	108, 109, 110, 111, 112
7. Epigenetic modifications	
7.1. Non‐coding RNAs	115, 117, 119
7.2. EZH2	115, 124, 125
7.3. DNA methylation	128, 130, 131
8. Tumor microenvironment factors	
8.1. Tumor endothelial cells	137, 138
8.2. Myeloid‐derived suppressor cells	141, 142, 144, 145
8.3. Cancer‐associated fibroblasts	148, 149, 152
8.4. Tumor‐associated macrophages	154, 155, 156
9. Glucose metabolism	159, 160

## MOLECULAR PATHWAYS ASSOCIATED WITH RESISTANCE TO TREATMENT WITH TYROSINE KINASE INHIBITORS

### Angiogenic pathway

Hypoxia is a hallmark of cancer, including RCC.^18^ Hypoxia increased the expression of the transcription factors HIF1 and HIF2.^19^ As mentioned in the introduction, HIFs are constitutively activated in ccRCC due to loss of the VHL gene. HIFs target several downstream genes, including VEGF, PDGF, IL6, IL8, transforming growth factor α, erythropoietin, epidermal growth factor receptor, hepatocyte growth factor (HGF) receptor (HGFR/cMET [mesenchymal‐epithelial transition]), placental growth factor, and fibroblast growth factor 2.^19^

In this sense, HIFs are a promising therapeutic target. Recently, an HIF2 inhibitor (belzutifan) was approved for VHL disease‐associated RCC.^20^ Hypoxia caused by the regression of tumor vasculature during TKI treatment may induce the expression of various proangiogenic factors that lead to TKI resistance.^21^ A recent review showed the role of hypoxia as a driver of tumor heterogeneity,^22^ which is a fundamental feature that hinders the efficacy of TKIs in RCC.^23^ Hypoxia also affects the tumor microenvironment (TME), which is a key factor of TKI resistance.^24^, ^25^ Combination therapy with an HIF2 inhibitor and cabozantinib is currently being assessed with the aim to maximally inhibit the HIF‐VEGF axis.^26^, ^27^

### Non‐angiogenic pathway

**FIGURE 1:**
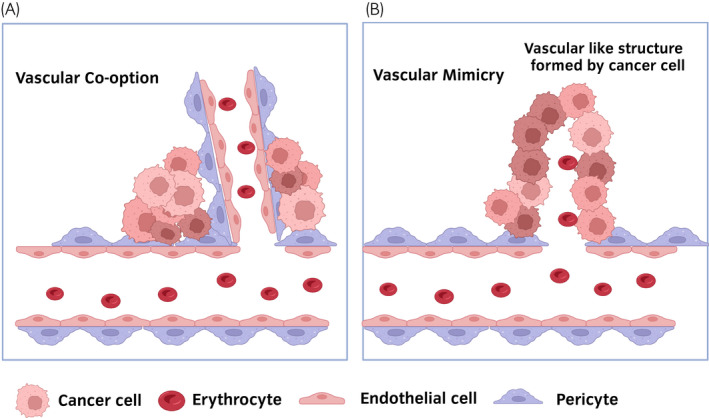
Tumor growth patterns associated with vascular co‐option or vascular mimicry. (a) Vascular co‐option. Cancer cells co‐opt pre‐existing mature blood vessels. (b) Vascular mimicry. Cancer cells form a vessel‐like structure.

The non‐angiogenic pathway is a relatively new theory in cancer biology.^28^ Accumulating evidence shows that tumors can progress without angiogenesis.^29^ The non‐angiogenic pathway is one of the causes of resistance to TKI.^28^ Vascular co‐option is a non‐angiogenic process whereby tumor cells can use the pre‐existing vasculature from the surrounding normal tissue (Figure 1a).^28^ A recent study showed that sunitinib treatment induced the switch from angiogenesis to vascular co‐option in a model of RCC lung metastasis.^30^ What is more, sunitinib treatment has limited efficacy in lung metastasis mediated by vascular co‐option compared with subcutaneously implanted tumors.^30^ Cancer can survive without angiogenesis through vascular co‐option, thus leading to TKI resistance.^31^

Vascular mimicry (VM) is another non‐angiogenic process whereby tumors generate vessel‐like structures without normal blood vessels or angiogenesis (Figure 1b).^28^ VM transformation allows tumors to become more aggressive.^28^ VM is associated with an aggressive phenotype and poor survival in ccRCC.^32^ A recent study showed that sunitinib treatment promotes VM transformation through estrogen receptors in RCC cells.^33^

### Epithelial‐mesenchymal transition (EMT)

EMT is a well‐studied process in which the phenotype is converted from polarized epithelial cells to mesenchymal cells through different molecular and biochemical changes. EMT plays an essential role in cancer biology, including resistance to chemotherapy.^34^ Sarcomatoid differentiation represents a histologic manifestation of EMT.^35^ RCC tumors with sarcomatoid differentiation are associated with poor prognosis and limited response to TKIs.^36^ A recent RNA sequence analysis showed that RCC tumors with sarcomatoid differentiation exhibit a highly proliferative molecular phenotype with relatively low angiogenesis.^37^ Some studies have shown that EMT markers, such as vimentin, twist, and snail, were upregulated in sunitinib‐resistant RCC cell lines.^38^, ^39^ Previously, we showed that a high EMT signature was associated with poor prognosis in mRCC patients treated with sunitinib.^40^ In a recent study, immunohistochemical analysis showed that the EMT phenotype was observed in human specimens from metastatic sites after sunitinib treatment. In contrast, tumor xenografts from metastatic sites showed not an EMT phenotype but a ccRCC phenotype. Xenografts from these tumors restored the sensitivity to sunitinib treatment.^41^ These findings indicate that EMT is associated with resistance to sunitinib. Although direct targeting of EMT is challenging, several drugs can indirectly inhibit EMT.^42^ A combination of indirect EMT‐inhibiting drugs and a TKI could potentially overcome EMT‐mediated resistance.

### Activating bypass pathways

**FIGURE 2:**
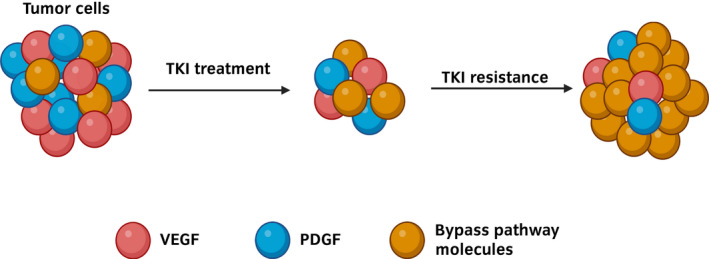
Proposed mechanism of activating bypass pathways in resistance development. TKI treatment initially reduces tumor size. Resistance to tyrosine kinase inhibitors can be mediated by activating bypass pathways. Sphere: tumor cell. Conglomerates of spheres: tumor. TKI, tyrosine kinase inhibitor.

In TKI‐resistant cancer cells, the bypass pathways are activated. Continued activation of bypass pathways leads to cell survival in the presence of TKI (Figure 2).^43^

#### Cytokines

Cytokines are involved in cancer development, progression, and drug resistance.^44^ IL‐6 is a soluble mediator with a pleiotropic effect on inflammation, immune response, and other fundamental biology.^45^ Recent studies showed that the expression of IL‐6 was increased after TKI treatment in RCC cell lines by RT‐PCR and ELISA analysis.^46^, ^47^ The inhibition of IL‐6 signaling by anti‐human IL‐6 antibody (tocilizumab) increased the efficacy of sorafenib treatment in RCC cell lines. Indeed, the combination treatment with tocilizumab and sorafenib significantly suppressed the tumor volume in vivo study.^46^ The IL‐6 neutralizing antibody also increased the efficacy of sunitinib treatment in vivo study.^48^ The prognostic value of IL‐6 has also been also analyzed. Two studies have shown that the high expression of IL‐6 was associated with poor prognosis in mRCC patients treated with sunitinib by immunohistochemical analysis and ELISA analysis.^49^, ^50^

IL‐8 plays a key role in the recruitment of neutrophils and other immune cells.^51^ The expression of IL‐8 was increased in the plasma of sunitinib‐resistant tumors compared with that of sunitinib‐responsive tumors, indicating that IL‐8 levels might predict clinical response to sunitinib.^52^ Treatment with IL‐8‐neutralizing antibodies resensitized the tumor to sunitinib in RCC in vivo model.^52^ Some studies showed that IL‐8 polymorphisms are associated with poor prognosis in mRCC patients treated with TKIs.^53^, ^54^

IL‐1α and IL‐1β constitute the members of the IL‐1 family. IL‐1β is a pro‐inflammatory cytokine.^55^ A recent study demonstrated that the combination with cabozantinib and anti‐ IL‐1β antibody significantly inhibited the tumor volume through the regulation of cytokines such as IL‐6, and TNF‐α in vivo study.^56^

NF‐κB transcription factor, which has an essential role in inflammation and immunity,^57^ was activated by sunitinib treatment. NF‐κB inhibition enhanced the efficacy of sunitinib treatment in RCC.^47^

#### PTEN/PI3K

PTEN acts as a negative regulator of the PI3K/Akt/mTOR pathway. Loss of PTEN leads to the constitutive activation of AKT/mTOR signaling.^58^ Although PTEN mutations are rare in RCC,^59^ PTEN plays an essential role in the biology of RCC.^60^, ^61^ One study reported that 786‐O cells, which have a PTEN mutation, were more resistant to sunitinib than other RCC cell lines, which contain wild‐type PTEN.^62^ Our previous study showed that knockout of PTEN by CRISPR‐cas9 induced resistance to sunitinib and sorafenib in RCC cell lines.^63^ Also, immunohistochemistry showed that the low PTEN expression group was associated with poor prognosis in mRCC patients treated with sunitinib or sorafenib as a first‐line therapy.^63^ Some preclinical studies have reported that PI3K inhibitors overcome sunitinib and sorafenib resistance in RCC cell lines.^62^, ^64^ Although clinical trials targeting PI3K/AKT in patients with advanced RCC have been tested to date, little anti‐tumor activity and excessive toxicity were observed.^65^

#### Fibroblast growth factor (FGF)/fibroblast growth factor receptor (FGFR) pathway

The FGF family plays an essential role in the biology of ccRCC.^66^ Among the FGF family members, FGF2, also known as basic FGF, was identified as the critical mediator of resistance to TKI.^67^ A recent study analyzed the expression of FGF2 and FGFR2 by immunohistochemical analysis in RCC.^68^ FGF2 and FGFR2 expression were observed in about 60% and 66% of RCC patients, respectively. Another study anayzed the expression of FGF2 by immunohistchemical analysis.^69^ The immunohistochemical analysis showed that negative, weak, and strong staining was found in 62%, 16% and 22% of RCC patients, respectively. Strong staining of FGF2 group was associated with poor prognosis after nephrectomy. FGF2 suppresses the anti‐angiogenic activity of sunitinib and activates phosphorylation of MEK and ERK signaling despite sunitinib being administered in RCC.^70^ In addition, PD173074, which is a potent inhibitor of FGF receptor activity, can overcome FGF2‐mediated resistance to sunitinib.^70^ These findings indicate that targeting of both VEGF and FGF2 signaling may be more efficacious than the use of sunitinib.^70^ Lenvatinib is a multi‐target TKI targeting VEGFR, FGFR, platelet‐derived growth factor receptor, RET, and KIT.^71^ So far, there has been no clinical trial directly assessing the efficacy of lenvatinib compared to sunitinib. However, a recent clinical trial showed that a lenvatinib and PD‐1 inhibitor combination improved overall survival compared to sunitinib monotherapy.^72^

#### Axl/GAS6

Axl is a member of the TAM (TYRO‐3, AXL, MER) tyrosine kinase receptor family and works with its specific ligand, growth arrest‐specific gene 6 (GAS6).^73^ High Axl expression is associated with shorter overall survival in RCC.^74^, ^75^ Several recent studies have shown that Axl/Gas6 signaling is involved in sunitinib resistance in RCC.^75^, ^76^, ^77^ Sunitinib targets VEGFR2, PDGFRβ, c‐Kit, and Axl.^78^ However, chronic sunitinib therapy (>2 weeks) increased Axl expression and induced EMT in RCC cell lines. Cabozantinib, which is a multi‐kinase inhibitor targeting MET, Axl, RET, and VEGF receptor,^79^ could overcome sunitinib resistance in RCC cell lines.^75^ Indeed, the two clinica trials (CABOSUN, METEOR) have shown the efficacy of cabozantinib. The CABOSUN clinical trial revealed that cabozantinib offers a significant clinical benefit compared to sunitinib as first‐line therapy in patients with mRCC.^80^ What is more, the METEOR clinical trial showed that cabozantinib treatment improved the overall survival of mRCC patients previously treated with TKIs compared to everolimus treatment.^81^

#### HGF/MET

MET (c‐met encoding) is a tyrosine kinase receptor. HGF is the ligand for the MET receptor and activates downstream signaling.^82^ High MET expression is associated with poor prognosis in RCC.^83^, ^84^ Some studies have shown that MET/HGF signaling is involved in the resistance to TKI in RCC.^85^ In mouse models, the expressions of HGF and MET were increased in sunitinib‐resistant tumors. The combination of sunitinib and a MET inhibitor significantly inhibited tumor growth in sunitinib‐resistant tumors.^86^ Cabozantinib, targeting multiple kinases including MET, could overcome sunitinib resistance in RCC.^75^ As I mentioned above, the recent two clinical trials (CABOSUN, METEOR) have shown the efficacy of cabozantinib treatment in first‐line treatment or second‐line treatment.^80^, ^81^

#### Angiopoietin/Tie

Angiopoietin‐2 (Ang2) binds to endothelial Tie2 receptor tyrosine kinase to regulate vascular development and maturation.^87^ Although the expression of Ang2 is low under normal homeostasis, it is increased in RCC.^88^ Ang2/Tie2 is involved in tumor growth and metastasis.^89^ The combination of sunitinib with an Ang2 inhibitor (trebananib) dramatically suppressed tumor volume in RCC.^90^ Plasma Ang2 was decreased at the beginning of treatment with sunitinib and then increased when sunitinib resistance appeared.^90^ Trebananib is an angiopoietin antagonist that neutralizes Ang1 and Ang2 interaction with the Tie2 receptor.^91^ A phase 2 clinical trial testing the combination between trebananib and sorafenib showed no significant improvement in survival,^92^ whereas another phase 2 clinical trial testing the combination between trebananib and sunitinib showed a potential benefit but with increased toxicity.^93^

### Transporters

**FIGURE 3:**
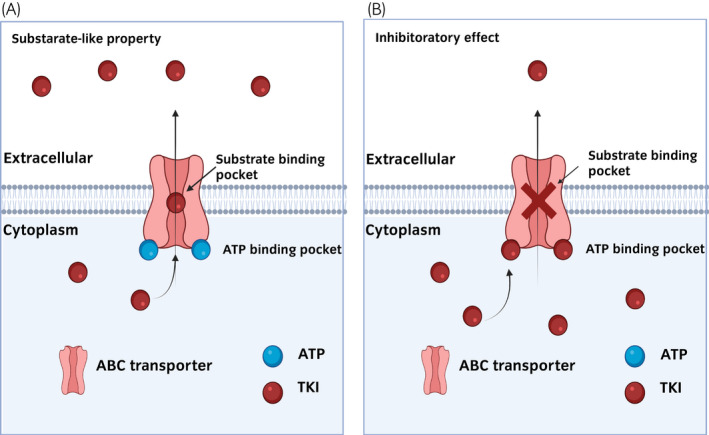
Transport of tyrosine kinase inhibitors (TKI) by ABC transporters. (a) TKIs function as a substrate property of ABC transporters. (b) TKIs inhibit the function of ABC transporters. TKIs bind to ATP binding pocket of ABC transporters.

The ATP‐binding cassette (ABC) and the solute carrier (SLC) membrane transporters play an essential role in the **translocation of many substrates, including TKIs, across membranes.** ^94^ ABC transporters primarily mediate the efflux of specific substrates,^95^ and SLC transporters are mainly involved in the influx or uptake of small molecules.^96^ Dysfunction of these transporters leads to inefficient drug concentration through increased efflux or decreased influx. Several studies showed that polymorphism in the ABCB1 gene was associated with poor prognosis in patients with mRCC treated with sunitinib.^97^, ^98^, ^99^, ^100^ A recent review showed that some TKIs have substrate properties and can bind to the substrate‐binding pocket of an ABC transporter, leading to TKI resistance (Figure 3a).^101^ Sorafenib serves as a substrate of ABCC2, which is one of the ABC transporters.^102^ Elacridar, an ABC transporter inhibitor, enhanced the efficacy of sunitinib treatment.^103^ However, some TKIs may also serve as inhibitors of ABC and SLC transporters (Figure 3b).^104^ These findings indicate that the combination of multi‐TKI treatment can enhance the function of the SLC transporter and simultaneously inhibit the function of the ABC transporter.^101^

### Lysosomal sequestration

**FIGURE 4:**
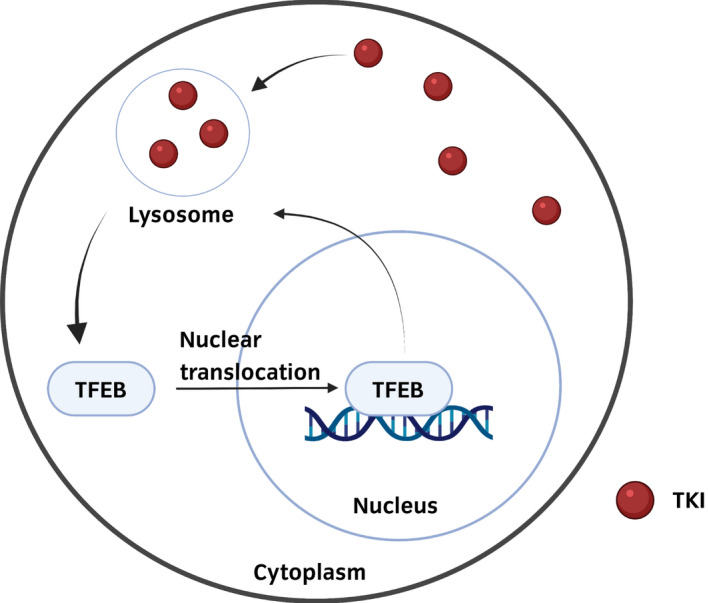
The schematic mechanism of lysosomal sequestration. TKI, tyrosine kinase inhibitor.

Lysosomal sequestration is a novel mechanism of resistance to several drugs.^105^ Lysosomes contain a variety of acidic hydrolases, which can degrade biological macromolecules.^106^ Lysosomal sequestration of drugs is a process in which the compounds with weak hydrophobic bases accumulate within lysosomes, preventing drugs from reaching their target.^107^ Some TKIs have biophysical properties of weak hydrophobic base molecules. Recent studies have shown that sunitinib and pazopanib were affected by lysosomal sequestration.^108^, ^109^ Intracellular sunitinib accumulation in acidic lysosomes was significantly higher in sunitinib‐resistant cells than in parental cells in RCC. However, the expression of phosphorylated Akt and ERK were comparable with that of parental cells despite sunitinib being administered. These findings indicate that the effect of sunitinib was reduced through lysosomal sequestration.^108^ Furthermore, the accumulation of sunitinib in lysosomes induced nuclear translocation of the transcription factor EB, which is a master regulator of lysosomal biogenesis.^110^ These findings indicate that an increase in lysosomes triggered by drugs further enhances lysosomal sequestration of drugs (Figure 4). A recent study showed that the lysosomal pathway was activated after sunitinib resistance.^111^ Although the involvement of lysosomes in resistance to TKIs increased interest in lysosome‐targeting strategies, there have been no reports on the clinical use of drugs targeting lysosomes.^112^ Basically, lysosomal targeting is not a cancer‐specific approach because lysosomes play a pivotal role in the biology of all types of cells.^113^

### Epigenetic modifications

Epigenetics modifications, such as DNA methylation, histone modifications, chromatin remodeling, and non‐coding RNA, can control the chromatin structure and influence gene expression without alterations in the DNA sequence.^114^ Epigenetic modification plays an essential role in cancer biology, including drug resistance.^115^

#### Non‐coding RNAs

A non‐coding RNA is an **RNA molecule transcribed from RNA but not translated into a protein.** ^116^ Micro RNA (miRNA), a particular class of non‐coding RNA, has been studied as a therapeutic target for TKI resistance in RCC.^115^ Although an increasing number of studies have reported the potential role of miRNAs for TKI resistance in RCC as reviewed in depth,^117^ there has been no reports of the clinical application of miRNA.^118^ One reason for the difficulty of drug development with miRNA is that it targets too many genes.^118^ miRNA has also been studied as a biomarker for TKI treatment.^119^ miRNA is relatively stable in various tissue specimens and body fluids,^120^ and the combination of various miRNAs has great clinical value as a biomarker. Some companies developed miRNA diagnostic panels for some cancer.^121^ Further studies are needed to identify the specific miRNAs related to TKI‐resistant.

#### Enhancer of zeste homologue 2 (EZH2)

EZH2 is a histone methyltransferase that methylates lysine 27 of histone 3.^122^ Overexpression of EZH2 enhances EMT by repression of E‐cadherin.^123^ EZH2 is one of the central epigenetic mechanisms of TKI resistance in RCC.^115^ Inhibition of EZH2 increased sensitivity to sunitinib in RCC cell lines.^124^ Furthermore, EZH2 can induce kinome reprogramming, leading to alternative pathways, such as increased tyrosine and serine phosphorylation.^125^ Immunohistochemical analysis showed that high EZH2 expression was associated with poor prognosis in patients with mRCC treated with sunitinib.^125^ Tazemetostat (an EZH2 inhibitor) is an FDA‐approved drug.^126^ Further clinical trials testing the combination of a TKI and tazemetostat are desired.

#### DNA methylation

DNA methylation is one of the essential epigenetic modifications in cancer.^127^ A recent study showed that the promoter region of glutaminyl peptide cyclotronsferase (QPCT) was hypo‐metylated.^128^ QPCT has glutaminyl cyclase activity and is involved in Huntington's disease.^129^ Overexpression of QPCT promoted sunitinib resistance through Ras/Raf/ERK signaling pathway.^128^ Some studies have shown the prognostic role of DNA methylation in TKI treatment.^130^, ^131^

### Tumor microenvironment factors

The TME comprises various components such as the tumor cells, extracellular matrix, fibroblasts, vascular endothelial cells, immune cells, and other stromal cells.^132^ TME‐mediated drug resistance results from crosstalk between the tumor cells and their surrounding stroma.^133^

#### Tumor endothelial cells (TECs)

The endothelial cells are an inner cellular lining that separates the circulating blood from the tissues. TECs play multi‐functional roles in tumor development and progression.^134^ The Notch ligand Delta‐like 4 (Dll4) is a Notch ligand. The Dll4‐Notch pathway regulates tumor angiogenesis and metastasis in cancer.^135^ Dll4 expression was increased in TECs of RCC compared to that in the normal kidney.^136^ The inhibition of Dll4 likewise inhibits the proliferation of TECs.^136^ One study showed that combination treatment with anti‐Dll4 and a TKI dramatically suppressed tumor growth in sunitinib‐resistant patient‐derived xenografts.^137^ Notch signaling is also involved in cancer stem cells in RCC.^138^, ^139^ Pharmacologic inhibition of Notch signaling promoted sensitivity to sorafenib in RCC cells.^138^ Several clinical trials targeting Notch have been assessed. So far, however, drugs targeting Notch have not been introduced into clinical use.^135^

#### Myeloid‐derived suppressor cells (MDSC)

MDSC are a heterogeneous group of immature myeloid cells.^140^ Sunitinib treatment suppressed the expression of MDSC in RCC.^16^, ^141^ However, intratumoral accumulation of MDSC was observed in sunitinib‐resistant RCC.^142^ Granulocyte colony stimulating factor (G‐CSF) is one of the key modulators of MDSC.^143^ G‐CSF recruited by tumors promoted resistance to TKIs by expressing various pro‐angiogenic factors.^144^ High expression of G‐CSF was associated with no clinical benefit in patients treated with TKIs.^145^ A recent study showed that anti‐G‐CSF treatment can induce protective tumor immunity in colon cancer.^146^ Studies on the effect of anti‐G‐CSF on TKI resistance are needed.

#### Cancer‐associated fibroblasts (CAFs)

CAFs can interact with multiple signaling pathways through paracrine mechanisms to promote cancer development, progression, and drug resistance.^147^ One study showed that tumors resistant to TKIs stimulated CAFs to secrete the pro‐angiogenic factor PDGF‐C, which generated angiogenesis and treatment resistance to TKIs.^148^ Sunitinib treatment increased the number of CAFs. Thus, an increase in CAFs can reduce the access of sunitinib to tumor cells, thereby leading to sunitinib resistance in RCC.^149^ Targeting CAFs is challenging because of the lack of a specific marker for CAFs.^150^ Histone deacetylase inhibitors can reduce the activation of CAF and eliminate CAF infiltration in the tumor stroma.^151^ A recent study showed that the combination of a TKI and histone deacetylase inhibitors overcame sunitinib resistance in RCC.^152^

#### Tumor‐associated macrophages (TAMs)

TAMs represent a major leukocyte population that infiltrates tumors.^153^ TAMs produce VEGF and other angiogenic proteins that may sustain angiogenesis and promote an environment of immunosuppression, which can lead to resistance to TKI.^154^ The high TAM group was associated with poor prognosis in patients treated with a TKI.^155^ However, a recent study showed that a high TAM signature was associated with improved survival in patients treated with TKIs.^156^

### Glucose metabolism

Tumor glucose metabolism is involved in the resistance to TKI treatment.^157^ The GLUT family facilitates the uptake of extracellular glucose.^158^ A recent study showed that sunitinib and axitinib treatment increased GLUT1 expression in extracellular vesicles, indicating that TKIs enhance the metabolic activity in RCC.^159^ Adding the glycolysis inhibitor 2‐deoxy‐d‐glucose to a TKI increased the sensitivity to pazopanib treatment in RCC.^160^

## CONCLUSION AND PERSPECTIVES

The treatment strategy for RCC is dramatically evolving. Indeed, several combination therapies with a TKI and immune checkpoint inhibitors have been introduced.^17^ Moreover, the combination of an HIF2 inhibitor and cabozantinib is currently being tested. These findings indicate that TKIs still exert an important influence on the treatment strategy of RCC.^26^ In this review, we discussed several mechanisms related to TKI resistance in RCC. Some molecules and drugs can restore the sensitivity to TKIs in preclinical analysis.

Recently, drug repurposing is becoming an attractive approach to lower drug development costs and shorten development timelines. As computational technology advances, the approach to drug repurposing is evolving.^161^ A recent review showed that several inhibitors of metabolism mitigate features of EMT.^42^ As we discussed, EMT is one of the causes of TKI resistance. We will need to further explore potential combination therapies using various approaches.

A large public database such as The Cancer Genome Atlas^162^ or the Gene Expression Omnibus^163^ has improved the understanding of the biology of RCC, and RNA sequence analysis has been performed in recent clinical trials to analyze molecular biology.^37^, ^164^, ^165^ However, data access and integration are the bottlenecks. There is a need for advanced approaches to integrate multi‐omics data. Further study is needed to improve our knowledge of TKI resistance and to develop successful treatment approaches that can overcome it.

## Author contributions

Yohei Sekino: Writing – original draft. Jun Teishima: Conceptualization; writing – review and editing. Gangning Liang: Conceptualization; writing – review and editing. Nobuyuki Hinata: Project administration; writing – review and editing.

## Conflict of interest

None declared.
